# On-Demand Wettability via Combining fs Laser Surface Structuring and Thermal Post-Treatment

**DOI:** 10.3390/ma15062141

**Published:** 2022-03-14

**Authors:** Deividas Čereška, Arnas Žemaitis, Gabrielius Kontenis, Gedvinas Nemickas, Linas Jonušauskas

**Affiliations:** 1 Femtika, Saulėtekio Ave. 15, LT-10224 Vilnius, Lithuania; arnas.zemaitis@femtika.com (A.Ž.); gabrielius.kontenis@femtika.com (G.K.); gedvinas.nemickas@femtika.com (G.N.); linas.jon@gmail.com (L.J.); 2 Laser Research Center, Physics Faculty, Vilnius University, Sauletekio Ave. 10, LT-10223 Vilnius, Lithuania

**Keywords:** femtosecond laser, laser texturing, laser fabrication, wettability, superhydrophobic, superhydrophilic, hydrophobic, hydrophilic, heat treatment, wettability transformation

## Abstract

Laser surface texturing (LST) is one of the surface modification methods that increase or provide new abilities for the material surface. Textured surfaces could be applied in different industrial areas to reduce wear and friction, promote anti-fouling, improve osseointegration, and other similar uses. However, LST is still in development and for reaching industrial level further optimization is required. In this paper, different metal alloy surfaces were fabricated with several patterns using the same laser parameters on each material and the results were compared. This could lead to possible optimization on the industrial level. Furthermore, research on the wettability properties of material and texture patterns depending on heat treatment in different temperatures was performed, showing complete control for wettability (from hydrophilic to hydrophobic).

## 1. Introduction

Femtosecond (fs) lasers are becoming more and more accepted tools in the industry. Due to their versatility, various light–matter interaction regimes can be induced [1]. This then can be used for both additive [2] and subtractive manufacturing [3]. One of the areas of interest for fs laser use is the possibility to induce highly controllable features on the surfaces of various materials for potential wettability control [4]. Overall, surfaces with controlled wettability properties can be produced in numerous ways, including various coatings or lasers with longer (for instance nanosceond) pulses [5,6,7]. Ultrashort pulse lasers offer a potential advantage of a higher degree of control of the thermal aspect of the process. In general, surface texturing was inspired by nature, observing living organisms’ interaction with the environment. This led to multiple studies providing research that shows this technology’s working principle and the development of ablation or texturing process [8,9]. By mimicking the surface topography of living organisms using laser surface texturing (LST) technology [10,11,12], material surfaces are modified by changing (increasing or decreasing) surface roughness, wettability [13,14,15] and adhesion properties. Modifications lead to multiple applications such as self cleaning [16], anti-icing [17,18], anti-fouling, antibacterial surfaces [19,20], wear and friction reduction [21,22], increased osseointegration [23,24,25], and more. However, to apply this technology at an industrial level, both a deeper understanding of the light–matter interaction as well as additional optimization aimed at simplifying the process is needed. One of the parameters to optimize are laser parameters used for fabrication, as it greatly affects the topography of the produced surface. Several studies were performed to investigate it, aiming at tying ablation results and laser parameters of different materials [26,27]. However, these studies do not address texturing results of different metal alloys. Furthermore, the surface features made by fs laser can have very different topographies, such as dimples, laser-induced periodic surface structures (LIPSS), grooves, or pillars. As wetting properties heavily depend on the topography itself [28,29], shape optimization is also extremely important. Therefore, alongside laser parameters used for fabrication, exact topography should also be optimized.

While fs radiation can produce various surface patterns, it also induces changes in surface chemistry. Because of this, after fabrications, laser-textured surfaces tend to be hydrophilic or even superhydrophilic. Only some time after laser exposure has passed and surfaces have been exposed to air or other media, wettability of the materials could become superhydrophobic [30,31,32,33]. Depending on the material, it sometimes requires a few weeks or even a month. This lengthens the time between fabrication and usage, which is undesirable in possible industrial applications, where it would result in additional storage and waiting-related expenses. To combat it, heat treatment in low (up to 250 °C) temperatures [34,35,36,37,38] could be used. When performed in the air, this increases the speed at which organic molecules from the air attache to the surface, leading to faster transformation to hydrophobic or even superhydrophobic surfaces. Faster transformation is necessary to apply LST technology for the industrial level.

The general LST field is quite expansive and, simultaneously, quite fragmented, making direct comparison between different materials and topographies difficult. Thus, in this paper, a direct comparison between surface features acquired using different fs laser parameters is performed. Additionally, produced surface features also vary from LIPSS and dimples to grooves and pillars. Industry-relevant metals, such as aluminum, steel, and titanium are investigated. Qualitative methods, such as optical images and profiles, as well as quantitative measurements of water contact angles, are provided. With subsequent discussion, a solid insight into how realistic fs laser surface structuring with subsequent heat treatment is for on-demand wettability control at the industrial level is given.

## 2. Materials and Methods

### 2.1. Materials

For topography experiments, several industry-oriented materials were used. The first ones are aluminum 2024 T3 and 7050 T74511. They both were acquired as disks, ∼49 mm in diameter and ∼5 mm high. Next, stainless steel PH13-8Mo and 17-4PH H1025 were tested. Samples of these materials were ∼47 mm in diameter and ∼5 mm high and ∼50 mm diameter and ∼4.9 mm height, accordingly. Finally, titanium Ti6Al4V was also tried. These samples were ∼59 mm in diameter and ∼5 mm high. For heat treatment effect on wettability, aluminum 2024 T3 and stainless steel PH13-8Mo samples were also supplied as 30 × 30 × 3 mm squares.

### 2.2. Samples Pre- and Post-Fabrication Treatment

At first, all samples were prepared for laser fabrication by cleaning them from oil residues and other foreign matter. Isopropanol at 99.8% was used for it. Each sample was rinsed in isopropanol and put in an ultrasonic bath for 10 min. After, samples were taken out from isopropanol and left to dry off for 15 min in ambient air. After fabrication, each sample was again cleaned by rinsing in isopropanol and put in an ultrasonic bath for 10 min. After a time, samples were taken out and left to dry off for 15 min in ambient air.

After drying, heat treatments were conducted in an electric oven in an ambient environment. Samples of topography testing were placed and heated at 200 °C for 2 h then taken out and let to cool down in ambient air for 15 min. Samples for heat treatment test on wettability were heated on single temperature and placed in a heated oven at 100 °C for 2 h. After this, they were taken out and cooled down in ambient air for 15 min. For other samples, temperature was increased by 15 °C, the whole process was followed in the same manner until 250 °C was reached.

### 2.3. Laser Fabrication

Four different patterns were produced on each sample. These were dimples, laser-induced periodic surface structures (LIPSS), grooves, and pillars. Each fabricated pattern was 10 × 10 mm in size. Fabrication was carried out using a Laser Nanofactory (Femtika) setup [39]. Primary light source—an fs Yb:KGW laser Pharos (Light Conversion). A laser beam was positioned using galvo-scanners and focused using an F-theta lens (focal distance—100 mm, spot size—∼27 μm).Polarization was linear and constant during all the processing. The whole process was controlled using 3DPoli software. Scanning strategy used during experiments was linear scanning. The only exception was dimple formation, here, the fabrication was performed by opening the shutter of a stationary beam for a fixed amount of time at a non-overlapping spot. Samples were fabricated in ambient air. Pressurized air was also applied to remove excessive debris and other leftovers by directly blowing them to the fabrication area.

### 2.4. Samples Observation and Testing

Samples were observed using optical microscope IX73 (Olympus) and by optical profilometer PLμ 2300 (Sensofar).

Wettability tests were performed after 15 min after cleaning (only for topography testing) and heat treatment by dropping distilled water droplets on the sample’s textured areas. Contact angles were measured using KSV CAM 200. The accuracy of measurement with this device is down to ∼2°.

After wettability, experimental water droplets were removed by tilting the sample to 90° and slightly shaking it. From some textures with high contact angle and low roll-off angle, water droplets roll off before reaching 90°. Nevertheless, on some textures’ surfaces water droplets hold on the surface even if the contact angle is high. Such an effect is called the rose petal effect. So, by slightly shaking samples, some amount of water drops off and only the residues on the attached area are left. Residue then evaporates in time in ambient conditions.

## 3. Results

### 3.1. Laser Parameters Effect on Materials and Alloys, Wettability, and Topography Testing

We began our work by determining the parameters needed to obtain distinct textures such as Dimples, LIPSS, Grooves, and Pillars on each material that could exhibit hydrophobic properties. Such types of textures were selected because they are periodical types and could be differently applied. Parameters were determined purely experimentally. The results are given in Table 1. Interestingly, to acquire a contact angle α greater than 120°, general parameters for all metals proved to be rather similar; this would also allow comparing the same type of texture topography between materials. It is a result of usage of fs pulses, which make processing relatively easy and highly replicable even for different topographies. Both wavelength and pulse duration could be maintained. The repetition rate also could be the same for 3 out of 4 cases—50 kHz. The only exception was LIPSS, as it benefited from an increased repetition rate. Dimples and LIPSS could also have been fabricated by a higher translation velocity of 0.5 m/s, while grooves and pillars required to reduce it five-fold to 0.1 m/s. However, grooves and pillars could have been made using quite a large scanning spacing—50 and 60 μm, respectively. Spacing for dimples was also relatively large at 80 μm. LIPSS, on the other hand, needed dense scanning of 4 μm. This can be explained by the fact that dimples, grooves, and pillars are relatively large features made by single non-overlapping scans (laser spot size used with 100 mm F-theta lens—∼27 μm), while LIPSS is a structure that appears at the surface by self-organization. Taking all of this into account, dimples proved to be the slowest surface texture to produce, with a rate of ∼0.74 cm^2^/min, with LIPSS, grooves, and pillars being relatively comparable in terms of manufacturing throughput at ∼1.2 cm^2^/min, ∼2.96 cm^2^/min, and ∼1.8 cm^2^/min, respectively.

**Figure 1:**
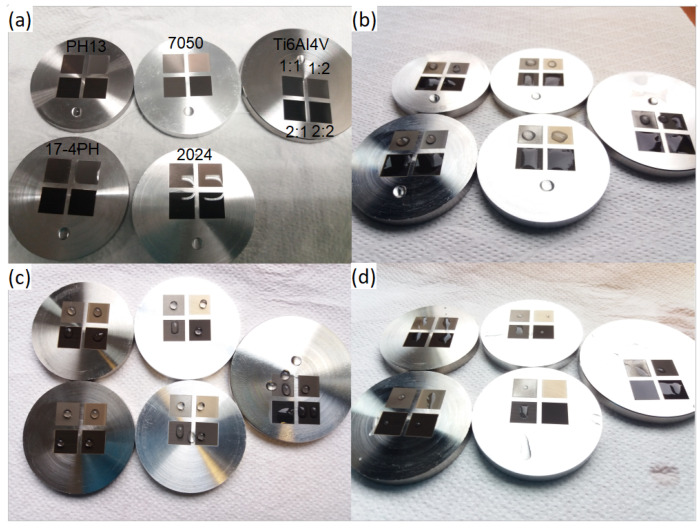
Wettability test of samples. (**a**)—post fabrication, (**b**)—post cleaning, (**c**)—post heat treatment, (**d**)—water residue. Codes for surface features 1:1—dimples; 1:2—LIPSS; 2:1—grooves; 2:2—pillars. Metals used are labeled in (**a**).

The next question was related to the post-processing of the samples. For it, a direct wettability comparison was made between before and after the second cleaning, shown in Figure 1a. Each sample maintains hydrophilic properties initially, but after cleaning in isopropanol Figure 1b only dimples and LIPSS maintains such properties. After 2 h of heat treatment at 200 °C Figure 1c, most samples again become hydrophobic. Comparing samples between alloys of metals, it seems that on a 17-4PH stainless steel sample fabricated textures after heat treatment show higher hydrophobicity properties than the B sample. In contrast, after heat treatment, only LIPSS textures show hydrophobic properties on the Ti6Al4V sample. Finally, water residue was observed to see if in time wetting properties change under the water drop Figure 1d. Results differed heavily material to material. Only 2024 aluminum samples with dimples and LIPSS had no water residue left. All other samples had some water left on them. It is interesting as initial hydrophobic properties seemed relatively comparable. Therefore, when considering hydrophobic properties, long-term (hours to days) performance needs to be evaluated. After checking the wettability of the textured areas after a substantial amount of time (about 10 month), the wetting properties of hydrophobic textures which were previously observed remained the same. Hydrophilic surfaces, however, became less hydrophilic or even hydrophobic.

**Figure 2:**
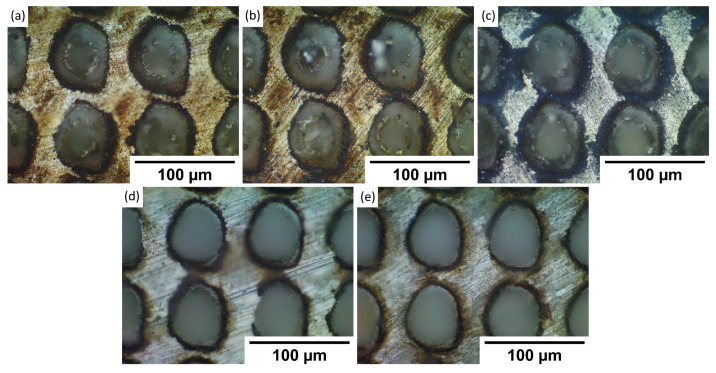
Microscope pictures, with ×50 magnification of each sample dimple texture. (**a**)—17-4PH stainless steel, (**b**)—PH13 stainless steel, (**c**)—Ti6Al4V titanium, (**d**)—2024 T3 aluminum, (**e**)—7050 aluminum.

**Figure 3:**
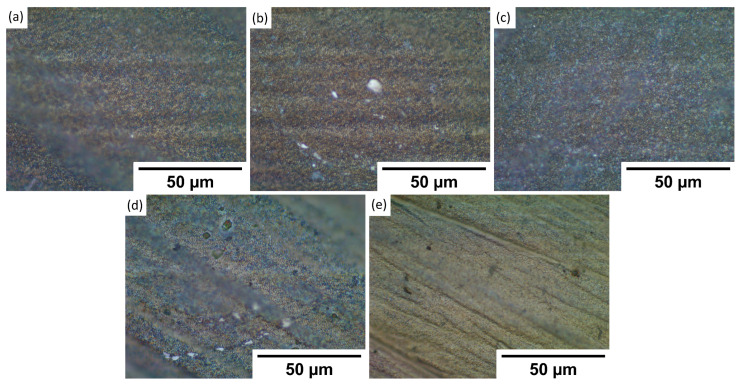
Microscope pictures, with ×100 magnification, of each sample LIPSS texture. (**a**)—17-4PH stainless steel, (**b**)—PH13 stainless steel, (**c**)—Ti6Al4V titanium, (**d**)—2024 T3 aluminum, and (**e**)—7050 aluminum.

**Figure 4:**
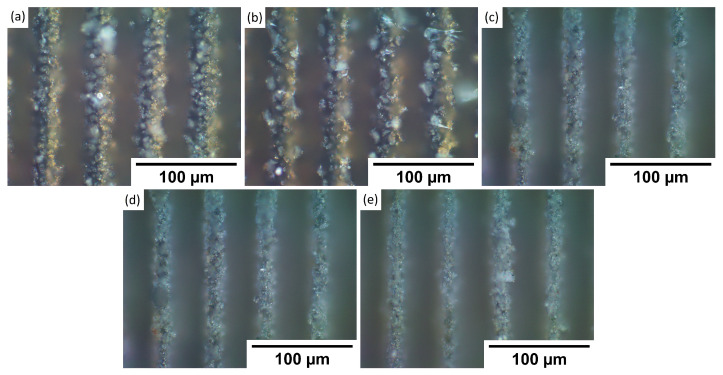
Microscope pictures, with ×50 magnification of each sample groove texture. (**a**)—17-4PH stainless steel, (**b**)—PH13 stainless steel, (**c**)—Ti6Al4V titanium, (**d**)—2024 T3 aluminum, and (**e**)—7050 aluminum.

**Figure 5:**
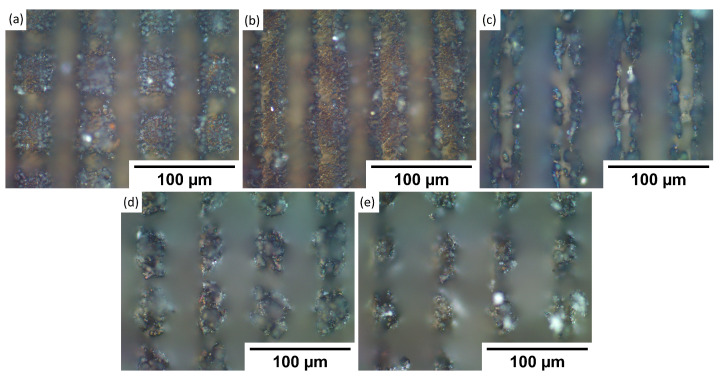
Microscope pictures with ×50 magnification of each sample pillar texture. (**a**)—17-4PH stainless steel, (**b**)—PH13 stainless steel, (**c**)—Ti6Al4V titanium, (**d**)—2024 T3 aluminum, and (**e**)—7050 aluminum.

To understand observed wettability properties and check if they depend on the topography of formed patterns, samples were investigated using an optical microscope. The goal was to see if maybe laser texturing of each metal has any substantial differences in produced patterns. By comparing textures in Figure 2, Figure 3, Figure 4 and Figure 5 to each sample, it was observed that there are no significant differences between stainless steel alloy dimples, LIPSS, and grooves textures. However, pillar textures proved the most difficult to compare using this methodology but seemed to not yield any significant differences. Furthermore, similar results are obtained between aluminum alloys on each texture. By comparing textures on titanium with other samples’ textures, it seems that structures are the most similar to stainless steel. Nevertheless, while there are minimal differences, generally shapes are quite comparable.

**Figure 6:**
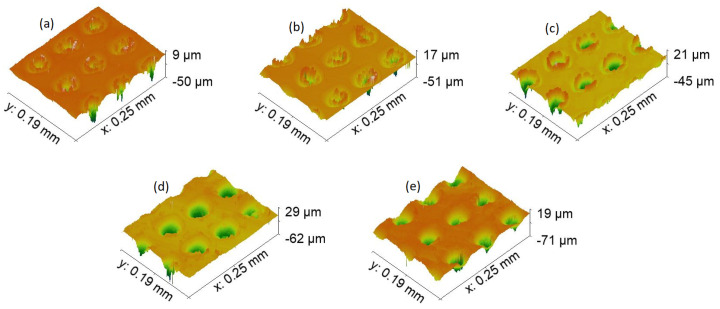
Topography, with ×50 magnification of each sample dimple texture. (**a**)—17-4PH stainless steel, (**b**)—PH13 stainless steel, (**c**)—Ti6Al4V titanium, (**d**)—2024 T3 aluminum, and (**e**)—7050 aluminum.

**Figure 7:**
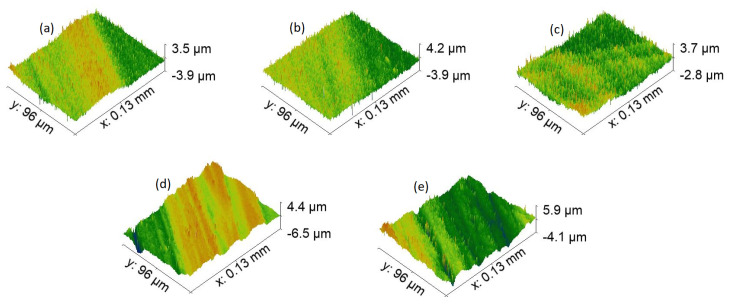
Topography, with ×100 magnification of each sample LIPSS texture. (**a**)—17-4PH stainless steel, (**b**)—PH13 stainless steel, (**c**)—Ti6Al4V titanium, (**d**)—2024 T3 aluminum, and (**e**)—7050 aluminum.

**Figure 8:**
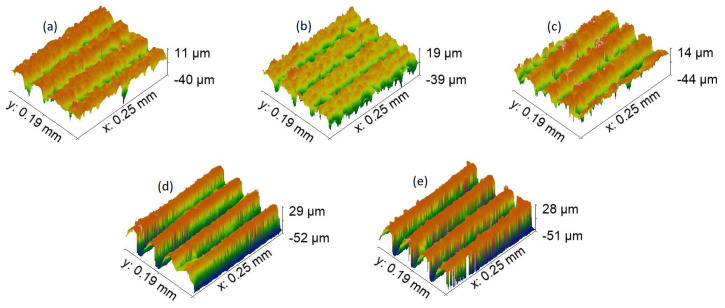
Topography, with ×50 magnification of each sample grooves texture. (**a**)—17-4PH stainless steel, (**b**)—PH13 stainless steel, (**c**)—Ti6Al4V titanium, (**d**)—2024 T3 aluminum, and (**e**)—7050 aluminum.

**Figure 9:**
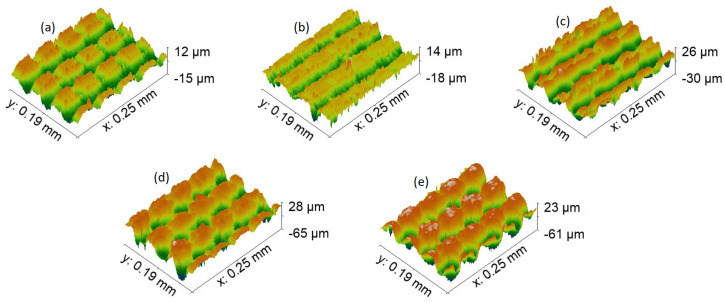
Topography, with ×50 magnification of each sample pillars texture. (**a**)—17-4PH stainless steel, (**b**)—PH13 stainless steel, (**c**)—Ti6Al4V titanium, (**d**)—2024 T3 aluminum, and (**e**)—7050 aluminum.

The next step was to compare topography in terms of the profile of formed features in Figure 6, Figure 7, Figure 8 and Figure 9. First, let us compare dimples in Figure 6. Their total depth is in the range from 59 μm for 7-4PH stainless steel to 91 μm with 2024 T3 aluminum. Dimples proved to be the deepest modification compared with LIPPS (which, as expected, were the shallowest), grooves, and pillars. Furthermore, both aluminum samples have dimples that are relatively deeper than steel or titanium. Aluminum samples also have substantially cleaner sides of the profile, while for harder materials some residue material is visible along the sides of the cut. This can be explained by the softer nature of aluminum, allowing easier removal of the material using a laser. A similar trend continued for all the other topographies. Interestingly, a potentially higher degree of residue material left after cutting also resulted in the pillars of PH13 in Figure 9a to be partially transformed back to grooves. However, the overall depth was still significantly different from the grooves of the same material (58 μm grooves vs. 32 μm pillars). Nevertheless, these differences seem to not have a direct correlation with wettability, neither in the short-term, nor long term. Thus, this clearly shows that while general surface topography can help in inducing wettability, it cannot achieve the required result alone.

On the other hand, surface chemistry also plays an important role in controlling sample wettability. One of the first sources of differences are chemical composition differences between materials and alloys themselves [40,41,42,43,44] as a result, various compounds are formed during fabrication. Several studies suggest [37,38] that after laser texturing material on its surface, active -OH groups could be formed, which means that surfaces after fabrication mostly are hydrophilic. During heat treatment, the surface starts to oxidize and the amount of -OH groups reduces, forming less active oxides that lower surface energy. At the same time, a small amount of organic and carbon compounds that could be found in air attaches to the surface and forms chemical bonds. This not only decreases surface energy but also surface polarity, so surface wettability shifts to hydrophobicity. So, by looking at chemical composition, heat-treated samples, due to attachment of organic compounds, maintain a higher amount of carbon on the surface, compared with samples right after laser fabrication. It is also possible that attachment of organic, carbon or other compounds could depend on the chemical composition of the material, leading to the formation of different compounds on material and wettability differences. At the same time, there is still a desire to somehow control it and/or induce it on demand.

### 3.2. Heat Treatment EFFECT on Wettability

**Figure 10:**
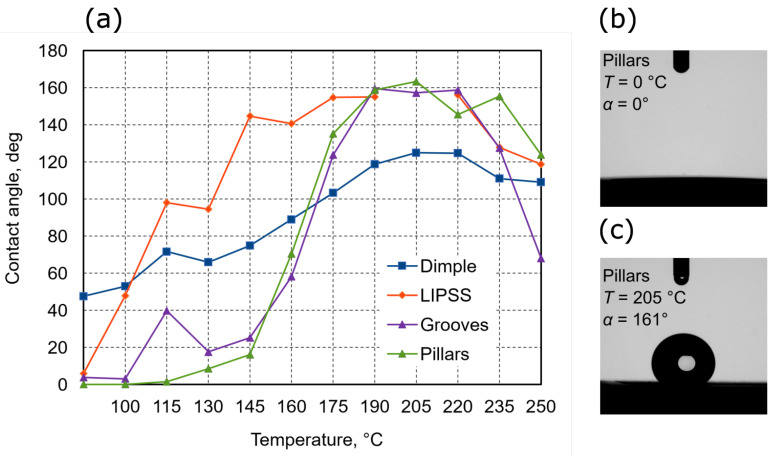
(**a**)—The contact angle of different patterns on aluminum surface dependence heat treatment in different temperatures. (**b**,**c**)—the lowest and highest acquired contact angle and temperatures needed to induce it.

**Figure 11:**
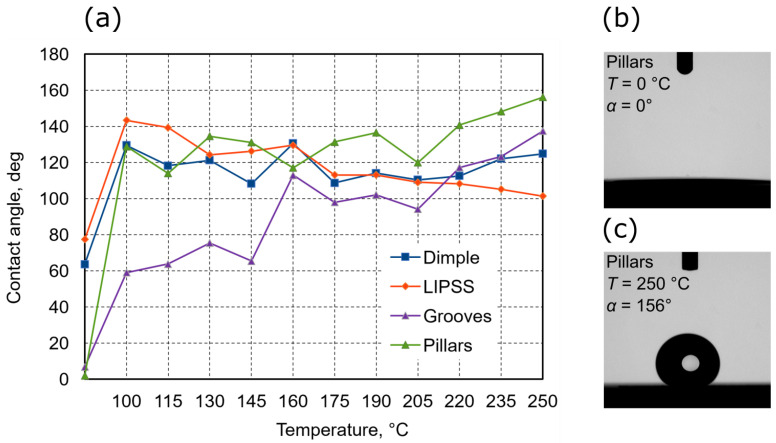
(**a**)—The contact angle of different patterns on steel surface dependence heat treatment in different temperatures. (**b**,**c**)—the lowest and highest acquired contact angle and temperatures needed to induce it.

Experimentation so far showed that surface features influence wettability, yet it cannot work without proper surface chemistry, while a laser can induce chemical changes via localized heating, it uses up precious laser exposure time. Thus, a much more suitable solution would be to use an auxiliary heat source and post-structuring bake. To prove it, steel and aluminum samples were heated to different temperatures and then induced wettability was measured. Steel and aluminum samples were chosen as these are the most relevant materials for fields of aerospace and shipbuilding. In these areas, contact angle on demand is especially important, as hydrophobic properties can be relevant for anti-icing or anti-fouling, while hydrophilic surfaces might be relevant for better paint adhesion. Results are shown in Figure 10 and Figure 11.

Aluminum-produced patterns initially are more hydrophilic. The contact angles of patters are: dimples—47°, LIPSS—6°, grooves—4°, and pillars—close to 0°. By heating samples in different temperatures, dimple patterns reach 125° contact angle at 205 °C, and in higher temperatures the contact angle starts to decrease. The LIPSS pattern reaches 155° at 175 °C and maintains a similar angle up to 220 °C. From 220 °C to higher temperatures the contact angle starts to decrease. Groove patterns reach 159° contact angle at 190 °C and maintain a similar angle up to 220 °C. After that, the contact angle starts to decrease in higher temperatures. Pillar pattern reaches 161° contact angle at 205 °C and maintains a similar angle up to 235 °C, after that the temperature contact angle starts to decrease. From all results, it seems that all patterns reach the highest contact angle and maintain it at a temperature between 190 and 220 °C. Furthermore, in higher temperatures all patterns start to decrease. From all tested patterns and obtain results, the highest contact angle provides grooves and pillars, LIPSS provides a slightly lower contact angle, and dimples the lowest. Overall, aluminum offers a lot more tunability using thermal post-treatment.

Same as aluminum, the contact angle of patterns on steel samples were measured. Without heat treatment, patterns on steel are also hydrophilic. Contact angles of patters are: dimples—64°, LIPSS—77°, grooves—7°, and pillars—2°. By heating samples in different temperatures, dimple pattern reaches 129° contact angle at 100 °C and then just maintains it in a range between 109 and 131°. LIPSS pattern reaches 143° at 100 °C and then decreases by increased temperature to 101° at 250 °C. The grooves pattern contact angle increased by increasing temperature from 60° at 100 °C and up to 137° at 250 °C. Pillars pattern at 100 °C reaches 128° contact angle and maintains the contact angle from 114° to 141° at 205 °C and then keeps increasing up to 156° at 250 °C. From all the results, it seems that each pattern reacts differently to heat treatment, and contact angles change depending on temperature. The grooves pattern contact angle only increases and possibly could reach even higher in temperatures higher than 250 °C. Pillar’s contact angle starts to increase more at 220 °C and could probably increase more in temperatures higher than 250 °C. However, the dimple pattern is quite stable and does not show a high increase or decrease in contact angle depending on temperature. Furthermore, the LIPSS patter contact angle only decreases in the provided results. It seems that the pillars and grooves patterns show the best hydrophobic properties and could be increased in higher temperatures. Furthermore, LIPSS pattern could also maintain a higher contact angle if heat treatment were done in lower temperatures. Interestingly, hydrophobicity was achieved almost immediately after heating patterns above 100 °C, showing that it is easy to induce on steel but cannot be tuned much. Furthermore, this experiment proves that patterns play an integral role in contact angle dynamics alongside surface chemistry.

## 4. Discussion

General surface properties, including wetting, are very important in a multitude of fields, including aerospace [45], maritime [46], heavy industry [47], and medicine [48]. At the same time, while various pilot-level tests are presented in the literature, there are still a multitude of challenges separating academic achievements from widespread use. These include longevity of surfaces, on-demand properties, and the possibility to achieve the required result in real-world environments. The work presented here shows some promise to address these challenges. Primarily, the temperature was shown to completely dictate wetting properties on demand. Additionally, as a single fs laser setup allows to acquire different surface features, this give an additional degree of freedom in tackling further challenges which might arise in developing the field of surface fictionalization.

Indeed, fs laser is in quite stiff competition against some other methodologies popular in surface functionalization. These include various coatings or other chemical methods [49] as well as induction of surface features using abrasive methods [50]. Chemical and coating methods surpass current fs procedures by sheer throughput, allowing them to coat up to square meter-sized surfaces in a matter of minutes. Abrasive methods are also very well established and can use already established industrial equipment, which do not require laser-based retooling. However, both of these methods lack the parameter control and flexibility of fs laser in terms of produced structure topography. Indeed, this work demonstrated that LIPSS, dimples, grooves, and pillars can be produced interchangeably. What is more, their profile and general topography are not random, as with most other methodologies. Finally, as shown in the presented results, post-processing using heating can expand these capabilities even further. It is an attractive addition, as heat treatment can be performed without an expensive laser and paralleled between multiple samples. Therefore, while fs-laser processing is still not very prevalent in industrial processing, there is a strong case to be made for it becoming a standard tool in the near future, either supplementing or completely replacing current chemical and abrasive methods.

Nevertheless, fs-laser processing still has some technical challenges inherent to the methodology itself. Throughput is the main one, while fs processing is extremely precise, its speed is bound to laser spot size and translation velocity. Indeed, it is a very widely discussed issue in both subtractive [51] and additive fs manufacturing [52]. In terms of speeding up laser surface processing, several approaches are possible. Multi-beam focusing is one of the most prevalent ones [53]. This is normally achieved using diffractive optical elements (DOEs) or other passive elements. The spatial light modulator is also an interesting possibility, as it allows unprecedented control of laser beam spatial distribution in the focal plane [54]. Scanning by applying acusto-optical deflectors is also a possibility [55]. At the same time, with possible drawbacks, fs processing brings some very distinct long-term possibilities. Ultrashort pulses allow very precise tuning of thermal effects during processing. This capability was expanded even more by the advent of fs bursts [56]. To date, it was shown to be suitable for such parameter-sensitive operations as polishing. However, one can imagine that one day such a degree of heat control can be used to induce surface thermal treatment during the main laser processing step, not require subsequent heat treatment, such as the one shown in this work. Furthermore, processing can be performed in various atmospheres, gas or liquid alike. This gives yet another degree of freedom to achievable surface chemistry, as the atmosphere can be either inert or even used specifically to create special chemical elements on the surface. Therefore, fs processing is simultaneously challenging and highly promising in the field of surface structuring.

## 5. Conclusions

Comparing each texture fabrication time found that dimples are the slowest to produce, with a ∼0.74 cm^2^/min structuring rate. Other textures, such as LIPSS, are ∼1.2 cm^2^/min, while grooves—∼2.96 cm^2^/min and pillars—∼1.8 cm^2^/min. So, the fastest way to fabricate the whole surface is by using a groove texture. Furthermore, by observing sample textures with a profilometer, it was noticed that differences between obtained structure heights on the same material alloys are quite small and are in tens of μm. From all comparisons, we could tell that the same or even slightly adjusted laser parameters could be applied for multiple alloys of the same material. Furthermore, by slightly optimizing parameters, the whole fabrication process could be easily shifted between steel and titanium, allowing quick adoption of different materials.

Heat treatment in different temperatures (at the beginning samples were treated at 100 °C and for other samples temperature was increased by 15 °C up to 250 °C) discovered that the optimal temperature to exhibit the highest hydrophobic properties on textured areas of steel is about 205 °C. Furthermore, steel allowed to achieve superhydrophobic surfaces with all the patterns faster and more consistently, while aluminum gave substantial tunability, especially with grooves and pillars (contact angle—from ∼0° to ∼160°). This enables to produce surfaces with certain wetting behavior by selecting texture type and required temperature on demand. From the hydrophobicity aspect in both materials, grooves and pillars provide the best results. Furthermore, in the aluminum case, LIPSS texturing also provides a high contact angle, while dimple texture the lowest. Even if dimple texture on material surfaces have less dense topography, it is not the fastest to fabricate and does not provide the best hydrophobic properties as other tested textures. Therefore, a compromise between throughput and application requirements should always be kept in mind.

## Figures and Tables

**Table 1:** Fabrication parameters of each pattern.

Laser Radiation Parameters	Dimples	LIPSS	Grooves	Pillars
Wavelength	1030 nm			
Pulse duration	500 fs			
Pulse repetition rate	50 kHz	250 kHz	50 kHz	50 kHz
Average power	2 W	1.5 W	5 W	7 W
Fluence	6.99 J/cm^2^	1.05 J/cm^2^	17.47 J/cm^2^	24.45 J/cm^2^
Pulse energy	0.040 mJ	0.0060 mJ	0.10 mJ	0.140 mJ
Pulse per spot	250 pulses	-	-	-
Open shutter time	5 ms	-	-	-
**Focusing parameters**				
Objective	100 mm F-theta telecentric lens			
**Scanning parameters**				
Scanning velocity	0.5 m/s	0.5 m/s	0.1 m/s	0.1 m/s
Scanning spacing	80 µm	4 µm	50 μm	60 μm

## Data Availability

Not applicable.
